# Diagnostic Interpretation of Non-Uniformly Sampled Electrocardiogram

**DOI:** 10.3390/s21092969

**Published:** 2021-04-23

**Authors:** Piotr Augustyniak

**Affiliations:** AGH University of Science and Technology, 30-059 Krakow, Poland; august@agh.edu.pl; Tel.: +48-697-032-858

**Keywords:** electrocardiogram (ECG) interpretation, non-uniform sampling, arbitrary sampling model, non-uniform patterns classification, non-uniform to time-scale transform

## Abstract

We present a set of three fundamental methods for electrocardiogram (ECG) diagnostic interpretation adapted to process non-uniformly sampled signal. The growing volume of ECGs recorded daily all over the world (roughly estimated to be 600 TB) and the expectance of long persistence of these data (on the order of 40 years) motivated us to challenge the feasibility of medical-grade diagnostics directly based on arbitrary non-uniform (i.e., storage-efficient) ECG representation. We used a refined time-independent QRS detection method based on a moving shape matching technique. We applied a graph data representation to quantify the similarity of asynchronously sampled heartbeats. Finally, we applied a correlation-based non-uniform to time-scale transform to get a multiresolution ECG representation on a regular dyadic grid and to find precise P, QRS and T wave delimitation points. The whole processing chain was implemented and tested with MIT-BIH Database (probably the most referenced cardiac database) and CSE Multilead Database (used for conformance testing of medical instruments) signals arbitrarily sampled accordingly to a perceptual model (set for variable sampling frequency of 100–500 Hz, compression ratio 3.1). The QRS detection shows an accuracy of 99.93% with false detection ratio of only 0.18%. The classification shows an accuracy of 99.27% for 14 most frequent MIT-BIH beat types and 99.37% according to AAMI beat labels. The wave delineation shows cumulative (i.e., sampling model and non-uniform processing) errors of: 9.7 ms for P wave duration, 3.4 ms for QRS, 6.7 ms for P-Q segment and 17.7 ms for Q-T segment, all the values being acceptable for medical-grade interpretive software.

## 1. Introduction

Compressed sensing was originally invented for speeding-up MRI scans. Consequently, sophisticated mathematical tools have been proposed in order to maintain the quality of the images reconstructed from partial k-space samples. This technique subsequently spread to other domains and numerous papers were published also on compressed sensing of ECG signals, partly replacing the earlier idea of ‘compression’. However, in addition to a new name and statistical background, the compressed sensing technique inherited compression flaws such as weak practical justification and no clear indication of the consequences to the medically-relevant content of the signals. Particularly, most of proposed methods compare locally downsampled signals to their original counterparts using general purpose error measures, and consequently neglect the temporal distribution of medical ECG content.

One of factors limiting the usage of non-uniformly sampled ECGs is the lack of procedures that could directly process the non-uniform signal to provide quantitative diagnostic outcomes or qualitative statements. Moreover, comparing diagnostic results calculated respectively from uniform and non-uniform ECGs is more relevant from the practical application viewpoint than comparing signal amplitudes in the time domain.

In this paper we review the classical diagnostic approach to the ECG interpretation and adapt its principal elements to the realm of non-uniformly sampled ECG records. The rest of this paper is organized as follows: In Section 2 we present standard chain of ECG interpretation procedures, in Section 3 we go through fundamentals of non-uniform sampling theory. Section 4 is divided into three parts related to heartbeat detection, classification and wave delineation (time measurement), in each we present uniform interpretation state-of-art approaches and propose solutions adapted to non-uniform ECG. In Section 5 we outline test signals in both uniform and non-uniform representations, taking an example of the latter one from perceptual studies on diagnostic data distribution. In Section 6 we present test results for all three interpretation procedures and Section 7 presents a discussion and concluding remarks.

## 2. ECG Diagnostic Procedures

Electrocardiography is a widespread diagnostic technique based on recording and analysis of phenomena related to electrical activity of the heart. It was invented at the beginning of 20th century [1] and further developed through decades in medical and technical aspects. The advent of digital signal processing brought numerous algorithmic approaches to the analysis of the ECG. Currently the ECG market is estimated to be worth USD5.8 billion, with a compound annual growth rate (CAGR) of 6.1%. Its most important part consists of long-term recorders (Holter), with CAGR of 12% [2]. A continuous demand for ECG market is propelled by common occurrence of health problems around the world, increasing number of ECG tests and systematic increase in geriatric population susceptible to heart diseases. Roughly estimating, one third of ECG tests are made remotely, i.e., in home care setup, what is supported by the advantage of true-to-life recording conditions, commodity of the patient and recently-by the need of social distance. This modality requires automated real-time review software and efficient communication with electronic medical record of the patient [3]. The number of recorders sold yearly worldwide can be estimated for 2.8 million and considering their life cycle, average duty cycle and a size of a rest-, exercise- and day-long records one easily can come to a total of 600 TB of data the ECG recorders daily deliver to healthcare system servers. Despite the size, all these data are easily manageable due to clear specification of Standard Communication Protocol for computer-assisted electrocardiography (known as EN1064:2020). However, the period of required data persistency, covering virtually all patient’s lifespan, justifies the research on effective yet transparent ECG storage format.

Either manual or automated, diagnostic interpretation of the ECG record is aimed to provide the cardiologist with roughly two kinds of information:

- Beat-to-beat variations of selected heartbeat parameters such as RR interval, beat types (arrhythmia), ST-segment, T-wave alternans etc., also known as sequence analysis.
- Details of the conduction path functionality, expressed in parameters of most typical heartbeat or group representative, also known as contour analysis.

For the purpose of providing this information, three procedures are mandatory components of the ECG interpretation software:

- a heartbeat detector,
- a heartbeat classifier, and
- a wave border delineator.

These three groups of procedures are usually developed and tested separately, according to specific, worldwide recognized testing tools and reference signals. A heartbeat (or QRS) detector identifies the presence of the ECG, the presence of heartbeats and their rhythmicity. The output of the detector is usually the information on approximate time coordinate of the fiducial point that subjects to further refinement. The detection result is used to calculate heart rate-related diagnostic parameters (such as heart rate variability, HRV) and as a reference for subsequent ECG signal segmentation and synchronization, that prepare beat data for classification. The classifier operates on signal segments surrounding the QRS in order to specify their likelihood and group them in clusters of similar features standing for particular types of normal or aberrant heart cycles. The number of clusters is representative to stimulus generation centers (physiological pacemakers) and conduction types (i.e., ways through the myocardium), while series of beats of various types form patterns of mutual conduction dependencies known as arrhythmias. Moreover, belonging to so called dominant class is a condition for inclusion of a beat to calculations of the HRV and ST-segment elevation series, which are meaningful in sinus rhythm only. Conversely, the percentage of beats in non-dominant classes stands for a marker of severity of possible conduction defect. Finally, for each heartbeat, or at least each cluster-representative beat, detections of P and T waves are made and positions of five points known as P-onset, P-end, QRS-onset, QRS-end and T-end are determined. These wave delimiters are further used for calculations of intervals in the heart cycle and to assess the stimulus conduction speed and repeatability. Precise delimitation of wave borders reduces the risk of confusing various conduction-related diseases when applying rules of automatic interpretation [4] and the accuracy of wave borders positioning is often considered as main quality factor of interpretive software. Despite cardiology standards allow for 10 ms inaccuracy (or 30 ms in a QT segment), some recently developed procedures show average errors of 1 ms or less.

The aim of this paper is to study the feasibility of devising procedures belonging to the above mentioned three groups and being able to directly process non-uniformly sampled ECG signal. What is more, we are also going to assess the extent of preservation of original diagnostic features in the non-uniform ECG representation and in the non-uniform interpretation process. As we used the MIT-BIH database [5] (publicly available under a US National Institutes of Health program) and the CSE Multilead Database [6] (commercially available), no recordings have been made in humans and no further ethical approval was required for this study.

## 3. Non-Uniform Sampling

Non-uniform sampling is a technique assuming each sample to be taken with different interval and amplitude scale. Fixing the amplitude scale simplifies the problem to the irregular sampling. In this kind of signals we can distinguish three cases:

- Uniform signals with occasionally missing samples, e.g., being results of data transmission or storage errors, where outliers are usually interpolated from the neighbor values.
- Partially decimated signals, where local sampling interval is a multiple of given basic interval, being usually a result of compressed sensing or time-domain reconstruction from incomplete dyadic wavelet representation; in these signals we assume the sampling interval to be given by a discrete-time function with discrete values.
- Signals with random sampling intervals; in these signals we assume the sampling interval to be given by a continuous time domain discrete value function.

In this paper we consider the third, most general variant of irregular sampling. Its mathematical foundations were developed by Aldroubi and Unser [7], who extended the classic (Shannon) sampling theorem, assuming that a given harmonic signal component could be locally reconstructed in an unambiguous way from its digital representation if and only if the local sampling interval is shorter than the period of its frequency. In their approach uniformly sampled signals are particular class of time series which, in general, are naturally sampled at variable intervals. This corresponds well to how we actually perform measurements in irregular sampling grids due to intrinsic or objective constraints.

The reconstruction condition was studied by Beurling [8], Landau [9], and collected by Aldroubi, Gröchenig, and Feichtinger into a consistent frames theory [10,11]. This theory was further developed by Chen [12], Benedetto [13], and others [14,15]. The frames theory is a generalization of orthonormal bases and Riesz bases concepts in Hilbert spaces. To introduce the main principle of frames theory one should recall that a given set of samples *X* = {*x_j_*} *_j_*_∈*J*_ ⊂ *R^d^* is sufficient to uniquely reconstruct function *f* if and only if *f* ∈ {*R^d^*} is bandlimited. Denoting *s_d_* the discrete-time signal as a function of time, *n*-the consecutive sample number, and *T*-sampling interval, the reconstruction is given as:

(1) $$s(t)=h_{T}*s_{d}(t)=\sum_{n=-\infty }^{+\infty }s(nT)\cdot h_{T}(t-nT)$$

and assumes that a continuous signal is a convolution of discrete-time regularly distributed coefficients and harmonic orthogonal Fourier basis in L^2^(−1/2, 1/2) given as:

(2) $$h_{T}(t)=\frac{\sin(\pi t/T)}{\pi t/T}$$

Similar reasoning for non-harmonic Fourier bases allows us to extend the Shannon sampling theorem for cases of non-uniform sampling. In this case the function also has to be bandlimited, but the local sampling density to guarantee stable unique reconstruction (known as lower Beurling density) must satisfy:

(3) $$D(X)=\underset{r\to \infty }{\lim}\underset{y\in R}{\inf}\frac{\#X\cap \left(y+\left[0,r\right]\right)}{r}$$

where ‘inf’ stands for ‘inferior’ denoting the lowest number of samples of bandlimited function *X* within the radius *r* around the sampling position *y*, and # stands for the power (i.e., set of all possible subsets) of the intersection of sets *X* and (*y* + [0, *r*]).

In practice, a solution to the irregular sampling problem consists in two tasks [10]:

(1) Given a generator *φ*, one need to find conditions on *X*, in the form of a density, such that for *c_p_* and *C_p_* being positive constants independent of *f* the norm equivalence holds:
  (4) cpfLvp≤∑xj∈Xf(xj)pv(xj)p1p≤CpfLvp
  Then, at least in principle, $f\in V_{v}^{p}(\phi )$ is uniquely and stably determined by ${\left.f\right|}_{X}$.
(2) One needs to design reconstruction procedures which are useful in practical applications to recover *f* from its samples ${\left.f\right|}_{X}$, when the norm equivalence (above) is satisfied. The numerical algorithm has to be efficient and fast, therefore first approaches useful for arbitrary sampling used adaptive weights to compensate for the local variations of the sampling density.

Gröchenig and Feichtinger proposed an efficient iterative projection algorithm [16,17] and later developed a set of algorithms [18] to convert signals between various irregular sampling grids including a regular grid as the specific case.

The reconstruction algorithm was initially meant to project signals between uniform and non-uniform sampling grids, but in practice it may also be used to transform signals from one non-uniform representation to another. Nevertheless, only one of them is optimal in the sense of redundancy reduction for given local bandwidth variability of the signal. In case of signals with highly-predictable bandwidth such as ECG, the arbitrary sampling profile is determined on a background of stimulus propagation physiology and tailored to the actual positions waves in the cardiac cycle (see Section 5.4). In case of ECG the irregular sampling combines concise discrete representation of the long baseline intervals and precise high frequency representation of relatively rare and short QRS complexes.

## 4. Proposed Methodology of Non-Uniform ECG Interpretation

### 4.1. QRS Detection in Non-Uniform Ecg Signal

The QRS detection has been probably the most referenced topic in computerized electrophysiology for a half of the century, therefore providing a complete review of the various methods in a single paper seems very difficult [19,20]. Most of the proposed software detectors are built accordingly to a two-step paradigm. In the first step a probability-like time series (referred to as *detection function*, DF) is built from the (multilead) ECG signal and in the second step this series is evaluated for representing the QRS or other components with use of a threshold usually of adaptive value [21]. An alternative paradigm assumes to translate the signal strip directly from the time domain to the decision domain (therefore referred to as ‘recognition’ instead of ‘detection’). A two-step paradigm requires calculations of DF *p*(*t*) that estimates the expectation of occurrence of the heartbeat at given time point *t*. To this point various approaches were proposed and can be roughly classified as: (1) transform-based (e.g., employing filtering or differentiation), (2) pattern matching-based (e.g., using cross-correlation or machine-state graph), (3) statistical or (4) artificial intelligence-based [22].

The pattern-matching methods are particularly suitable to work well with non-uniformly sampled signals. They do not assume regular distribution of points in the time axis and use sampling-invariant approximation to best fit patterns to the real signal [23]. Given high definition (i.e., nearly analog) section like the QRS projected to various non-uniform grids can be found best matched to nearly the same pattern and detected in a repeatable, i.e., grid-independent way, what is not possible with tools like filtering, transforms, or Artificial Neural Networks, and hardly feasible with statistical approaches. The simplest approximation figure, the straight section is probably not useful for QRS detection; therefore we focused on the second simplest figure, the isosceles triangle. A group of detection methods called angular methods, originated from the analysis of the angle between the legs (i.e., triangle sides of equal length) best fitted to the ECG signal.

In 2010 Martinez et al. proposed a complete detection and delineation algorithm based on phasor transform [24]. The phasor is a complex number with magnitude and phase and in this particular implementation each sample of the ECG was transformed to a pair of real component of constant value and imaginary component dependent on ECG amplitude. This method is particularly useful since instantaneous phase variations in phasor ECG representation points are robust to local amplitude changes and thus similar approach can be applied to QRS, P, and T-waves delineation.

Analysis of the R-wave angle was proposed by Romero et al. [25] to detect the morphologic QRS changes typical to acute myocardial ischemia. They found changes of the upstroke and downstroke angles (respective Q and S waves) to be a sensitive marker for detection of the ischemia (upstroke angle is negative and falls while downstroke angle is positive and rises), whereas R-peak angle significantly correlates with the amount of ischemia.

In 2015 Song et al. presented a mature method of single lead real-time heartbeat detection using the geometric angle between two consecutive samples of the ECG signal [26]. Their method consists of three steps: (1) elimination of high-frequency noise, (2) calculation of the angle at given point of ECG signal being prospective candidate to R-peak, and (3) detection of R-waves using a simple adaptive threshold-based technique.

Based on similar idea we proposed a regression-based sampling-invariant method to detect QRS complexes in irregularly sampled ECG signals [27]. The cloud of samples falling in a given time range Δ*t* is expected to include at least two points and was approximated by a line segment with given slope value *m* using nonparametric regression estimation technique (known as Nadaraya-Watson approach [28,29]). Calculating *m*(*t*) for each time interval Δ*t* moving in regular time steps *t* generates regular time series with sampling interval independent of ECG sampling variations. Various lengths and moving steps of Δ*t* have been studied to provide optimal accuracy and positive predictive value [30].

**Figure 1:**
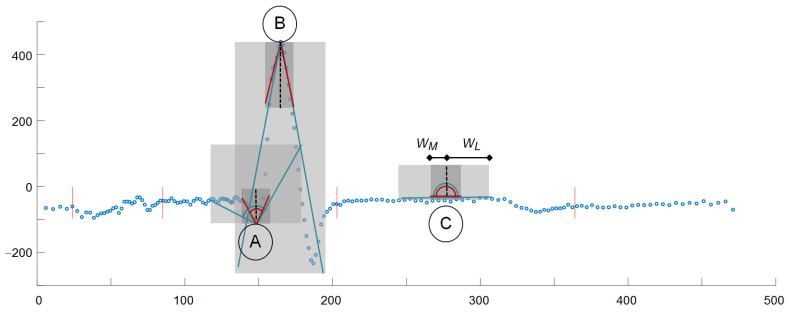
Working principle of the proposed QRS detector for non-uniform ECG signals (File CSE_MA_001, lead aVL). At the beginning of QRS (**A**) M (red) is small but L (blue) is large, at the peak of QRS (**B**) both M and L are small, and at the rising slope of T (**C**) both M and L are large.

We adopted a two-step approach consisting in building a DF and then thresholding its values. Since at the stage of detection, local distribution of information in the analyzed ECG is not known, the most reasonable is using the DF with equidistant samples. This approach makes the sampling of the DF independent of ECG sampling. To calculate the value of DF at each new time point (Figure 1):

(1) We build two pairs: (1) a long time (Δ*t_L_* = 61 ms) and (2) a short time (Δ*t_M_* = 20 ms) of triangle legs with ends *w_L−_*, *w_L+_*:
  (5) $$w_{L-}=\left(t-\Delta t_{L},\;t\right),w_{L+}=\left(t,\;t+\Delta t_{L}\right)$$
  and *w_M−_*, *w_M+_*:
  (6) $$w_{M-}=\left(t-\Delta t_{M},\;t\right),\;w_{M+}=\left(t,\;t+\Delta t_{M}\right)$$
(2) We determine the slope coefficients *m_L−_*(*t*) and *m_L+_*(*t*) (and respectively *m_M−_*(*t*) and *m_M+_*(*t*)) of segments *y* = *m·t* best fitted to the selected samples as:
  (7) $$m_{t}=\frac{\sum_{j=1}^{J}K_{j}\cdot \left(t_{j}-t\right)\cdot v_{j}}{\sum_{j=1}^{J}K_{j}\cdot \left(t_{j}-t\right)}$$
  where *J* is the total number of samples in the window, *j* is the current sample number relative to the window onset, and *K* are weighting window coefficients. The weighting coefficients are used to differentiate the influence of samples depending on their distance to the cloud center. In the experiments initially we used flat window (i.e., all points equally important), but finally applied Gaussian window of type $K_{j}=\frac{1}{\sqrt{2\pi }}e^{-j^{2}/2}$.
(3) We calculate the angle *L*(*t*) of best fitted triangle legs *w_L−_* and *w_L+_* adjacent to the time point *t* (Equation (8)) as in [26]:
  (8) $$L\left(t\right)=\tan^{-1}\frac{m_{L+}\left(t\right)}{m_{L-}\left(t\right)}$$
  and reuse (Equation (8)) to similarly determine the angle *M*(*t*) of best fitted triangle legs *w_M−_* and *w_M+_*.
(4) Since the heartbeat peak is characterized by significant turn of activity represented in small values of angles between long time triangle legs and concurrently between short time triangle legs, we simply take the inverse of product of these values:
  (9) $$p\left(t\right)=\frac{1}{L\left(t\right)\cdot M\left(t\right)}$$
  or, in case of a multilead record:
  (10) $$p\left(t\right)=\frac{1}{\sqrt{\sum_{c=1}^{C}{\left(L\left(t\right)\cdot M\left(t\right)\right)}^{2}}}$$
  where *C* is the total number of leads and *c* stands for the current lead number.

The time of heartbeat occurrence is determined as upward crossing of *p*(*t*) and a specified threshold. As most of methods reported in literature, we use the adaptive threshold. During the initialization, the procedure calculates the initial threshold value based on detection function in a given time window (e.g., 2 s) as in [21]. During the analysis of subsequent signal the threshold value *H*(*t*) is modified either at the presence of local maximum of DF or unconditionally at time point *t* accordingly to Equation (11):

(11) $$\left(t\right)=0.9\cdot H\left(t-N\right)\;+\;0.1\cdot \frac{1}{N}(\sum (n_{o}:n_{o}>H)-\sum (n_{u}:n_{u}\leq H))$$

where *N* is the number of DF samples in the time interval and *n_o_* and *n_u_* are values of DF falling respectively over and under the threshold. The duration of time interval, being a compromise of the adaptation responsiveness between required reaction time and suppression of overshoots, was set empirically to 120 ms.

The output information produced by thresholding operation is the time coordinate *t* of newly detected beat or 0 otherwise. The value of *t* is not expected to precisely indicate a fiducial point in the signal (e.g., R-wave peak), but must be unique per heartbeat to avoid false positive detections.

### 4.2. QRS/Beat Classification for Non-Uniform Patterns

Heartbeat classification is probably the second most exploited area in the domain of automated ECG interpretation. Justification of this is twofold: firstly, because the conduction pathway in the heart (manifested itself in the QRS shape) is representative to several diseases, secondly, because the number of heartbeats in long-time records easily reaches 100 thousand, what limits the chance of reliable detection of a single conduction aberration by non-automated approaches. Moreover, to reduce the computational burden of the whole interpretation process, usually only the heartbeats representatives to the classes (clusters) are subject to delineation and further contour analysis.

As several reviews of the ECG classification show, the problem can be first decomposed to: (1) clustering and (2) cluster morphology identification. The latter is performed with assumption that clusters are homogenous and with respect to the cluster representative heartbeat. Moreover, proper morphology detection requires delineation of waves, described later in Section 4.3. The clustering itself can be defined by (1A) the distance metric applied and (1B) the clustering method.

Two groups of strategies are used to define the degree of similarity of two representations of heartbeats in the ECG employing either the amplitude- or the feature-based distance. Although amplitude-based distance is easier to manage (e.g., the tolerance is easier to refer to real units) and calculation of features needs additional computational expenses, starting from few hundreds of heartbeats clustering with features is usually less expensive. Several proposals of features can be found in literature [31,32,33,34], in particular: descriptors based on wavelets [35,36,37], cosine transform [38], principal components analysis [39], independent component analysis [40], higher order spectra (HOS) [41,42] and several amplitude value derivatives.

The clustering method (classifying engine) can employ one of several proposed methods such as linear discriminant (LD) [43], AdaBoost [44], multilayer perceptron (MLP) [45,46], genetic algorithm-back propagation neural network (GA-BPNN) [47], convolutional neural networks (CNN) [48,49], and support vector machine (SVM) [50]. Otherwise, an ensemble of classifiers may be applied to integrate the outcome of multiple classifiers working with feature subsets [51,52,53]. A review of machine learning techniques for ECG classification can be found in [54].

Since the feature set characterizes the heartbeat in assessment of its likelihood to other beats in the ECG record, adequate selection of features is of primary importance. Desired features should be discriminative for most frequent beat morphologies, but additionally sampling-invariant, to comply with the non-uniform data representation. After initial review of features we did not found any candidate to show sampling model invariance and consequently used the amplitude as the best parameter to clearly express differences between signals.

The proposed implementation of distance metrics between two sections of non-uniformly sampled signals is based on a graph representation of the signal and graph similarity measure. In the graph representation a strip of non-uniform signal of given duration is described as nodes with value and time attributes and edges with length and slope attributes. Since edges and nodes are mutually dependent and the duration of the strip is constrained, there is a coupling between edge and node similarity scores. An iterative algorithm determining the similarity score was invented by Kleinberg [55], but later alternative methods like similarity flooding [56] and SimRank [57] were proposed. This topic received a big attention of researchers, particularly in the pattern matching and data mining communities [58]. The Kleinberg algorithm consists in determining the normalized similarity (i.e., 1 means ‘identical’ and 0 means ‘totally different’) between a given node *i* in graph GA and any node *j* = 1…*J* in graph GB and summarizing the scores for each node of GA. The same has been proposed for edges and integrated to a graph matching result.

Let *x_i,j_*(*k*) stand for similarity of node *i* in GB and node *j* in GA at stage *k* then [58]:

(12) $$x_{i,j}\left(k\right)=\underset{r:\left(r,i\right)\in E_{B},s:\left(s,j\right)\in E_{A}}{\sum }x_{r,\;s}\left(k-1\right)+\underset{r:\left(i,r\right)\in E_{B},s:\left(j,s\right)\in E_{A}}{\sum }x_{r,s}\left(k-1\right)$$

Similarly, if *y_p,q_*(*k*) denotes the edge similarity score between edge *p* in GB and edge *q* in GA then:

(13) $$y_{p,q}\left(k\right)=x_{s\left(p\right)s\left(q\right)}\left(k-1\right)+x_{t\left(p\right)t\left(q\right)}\left(k-1\right)$$

and consequently, the iterative matrix notation of similarity score *s_k_* is:

(14) $$s_{k}\equiv {\left[\begin{array}{c} x\\ y \end{array}\right]}_{k}\leftarrow \left[\begin{array}{cc} 0 & G^{T}\\ G & 0 \end{array}\right]{\left[\begin{array}{c} x\\ y \end{array}\right]}_{k-1}$$

An alternative approach to define graph matching uses an assignment matrix built for any combination of nodes in both graphs [59]. Neglecting the edges affects the distance linearity, but maintains the monotonicity due to edges and nodes similarity coupling mentioned above. Shall one of the graph be a sub-graph of the other (as in case of decimated signals), only the corresponding node values would subject to similarity evaluation (with some zeroes for nodes with missing counterparts). Unfortunately, we have to assume random sampling interval and therefore graph nodes are attributed with time and amplitude. The evaluation of graph similarity will then consist of two processes: (1) find node correspondence of minimum time difference (assuming it will be a mixture of one-to-many and many-to-one assignments) and (2) calculate the amplitude distance. Otherwise, one can calculate amplitude values by interpolating GA node values in time points of GB and cumulate the absolute difference of values as the distance score. The latter approach allows us to apply additional weighting factor to modulate the similarity score depending on the time attribute of the node.

In this approach the assignment matrix *M* between nodes *i* = 1…*I* in GA and nodes *j* = 1…*J* in GB may be denoted as [59]:

(15) M=   1    2      3    4  ijd1,10000d2,2000d2,30000d3,4d4,41234

where assignment A(1)↔B(1) is of one-to-one type, samples B(2) and B(3) were both found closer (in the meaning of time) to A(2) (one-to-many assignment), and samples A(3) and A(4) were both found closer to B(4) (many-to-one assignment). Consequently, the similarity score *s* has been proposed as:

(16) $$s=\sum_{i=1}^{I}\sum_{j=1}^{J}d_{i,j}$$

Davenport et al. [60] proposed direct inference methods (for signal classification among others) based on compressive measurements, without the need of reconstructing the uniform signals. Wimalajeewa et al. [61] provided theoretical studies on performance of signal classification. They derived formulas and showed examples how the dimensionality reduction in compressive sensing affects the classification performance in comparison to classification results with the original uniform signals. They also proposed a Chernoff distance [62] between two probability density functions as classification tool probably the most robust to sparsity increase. More recently, Wimalajeewa and Varshney [63] studied the Bhattacharya distance [64] and found that its ability to represent inter-signal correlation in compressed domain depends on similarity of sampling grids used to compress data at particular sensors. Alternatively, Cleju [65] proposes an arbitrary compressed sensing acquisition matrix based on nearest correlation between dictionary atoms. This approach would particularly be close to the arbitrary model-based ECG sampling, however, due to beat-to-beat variability, we cannot assume that consecutive beats were sampled at similar points.

**Figure 2:**
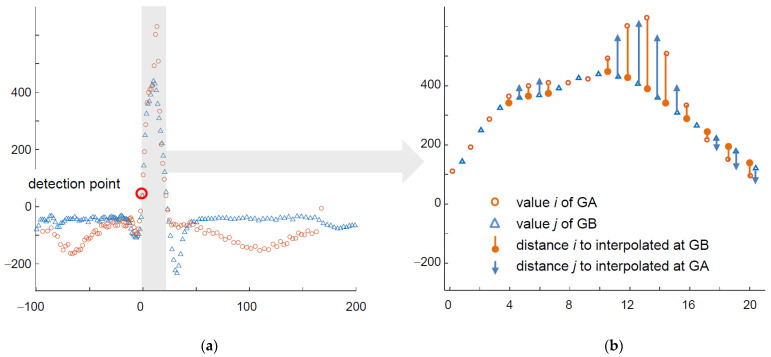
Principle of the similarity score calculation for non-uniform patterns (**a**) whole heartbeat, (**b**) magnified QRS region.

Considering all state-of-art ECG classification methods and general rules of non-uniform patterns classification, we proposed a custom method adapted to arbitrary ECG sampling based on graph representation of the signal and dissimilarity measure. The algorithm starts with converting all samples of the heartbeat (i.e., in a time window starting at 200 ms before the beat detection point and ending at 400 ms after it) to a sequence of pairs (value, time), where the time is relative to the detection point and monotonic (Figure 2).

- In the first step, for each node in GA corresponding node(s) in GB are found so as they have most similar time attribute; similar correspondence is reciprocally built for GB nodes.
- The GA node at the detection point is aligned to its counterpart at the GB detection point.
- For each other node the node of minimum absolute time is selected and the value of its counterpart node (i.e., with the same time attribute) is interpolated in the other graph.
- Similarity score is then calculated as cumulative sum of absolute differences of values at subsequent nodes modulated by a time-dependent weighting factor.
- Dividing of the similarity score by the average amplitude ends calculation of the distance.

The algorithm above works accurately, but is computationally expensive and needs much time to calculate similarity score between heartbeats, in particular in long-time multilead records. Therefore two simplifying assumptions were taken into account in the practical implementation:

- In a multilead record common wave border values are used to determine arbitrary sampling grid at signal source, therefore time attributes of graph signal representation in particular leads are the same.
- In arbitrary sampling shorter sampling interval was applied to more informative parts of the ECG (e.g., within the confines of QRS), consequently the information on sampling interval may be used as modulator of the similarity score.

**Figure 3:**
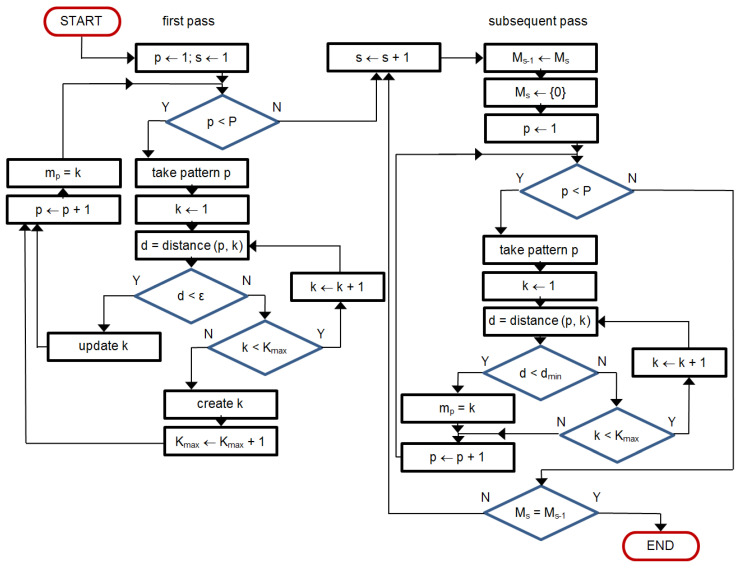
Multipass classification scheme (simplified).

The classifying engine is a multipass iterative process with two stop conditions: (1) the same beat assignments in two consecutive passes and (2) maximum iteration count achieved (Figure 3).

**Figure 4:**
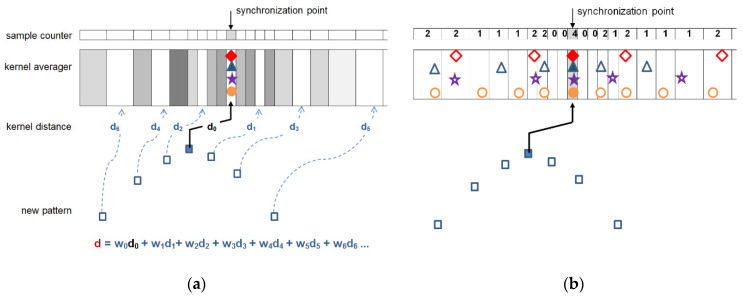
(**a**) Comparison of the non-uniformly sampled beat with the content of arbitrary class kernel; (**b**) arbitrary kernel pattern update by a newly accepted beat.

In the first pass an empirical similarity threshold is applied on measured beat-to-kernel distance (Figure 4a) to decide whether the currently processed beat (1) falls in one of the existing classes or (2) creates a new class. In case 1 the class kernel is modified (Figure 4b) in case 2 the ‘founding’ beat temporarily becomes the (non-uniform) class kernel. At the end of first pass the class assignment is memorized for future comparison, and subsequent pass begins.

At the beginning of each subsequent pass class kernels are maintained but the beat assignment is forgotten. Instead of the threshold used in the first pass, beats are now expected to be assigned to the class of the nearest kernel. Kernels are not modified until the end of each pass, and then the assignment achieved is compared to the attributes memorized from previous pass. In case of identical assignment the classification is completed, otherwise, last class attributes are memorized, class kernels are updated based on all member beats and the classification restarts.

Two more remarks were taken into account in our practical implementation:

- In threshold-based decision making the beat-to-kernel comparison can be stopped immediately when the cumulative dissimilarity exceeds the threshold value; doing so helps to avoid unnecessary calculations.
- While in uniform case updating of the kernel may be done by averaging of respective samples, arbitrarily sampled beats may update the kernel either by updating the values existing near to respective time coordinates, or by contributing with new data falling between the existing samples. Therefore, for class kernel representations we adopt a beat-independent arbitrary quantization pattern (Figure 4). It increases local sampling density (i.e., decreases the sampling interval) near the fiducial point to a value 8-times higher than maximum signal sampling density and decreases it respectively with the distance from the fiducial point. Consequently, amplitude values next to the fiducial point (i.e., in central part of the pattern) contribute more significantly to the likelihood assessment than the peripheral part of the beat or kernel. This significantly limits the count of required interpolations and saves much of the computation burden at the price of classification accuracy.

### 4.3. Delineation of QRS Waves in Non-Uniform Representation

ECG waves’ delineation is probably less represented in literature as a research topic, however, due to paramount influence the accuracy of wave borders has to the reliability of qualitative diagnostic outcome, it is known as principal area of competition between interpretive software manufacturers. The portfolio of methods proposed for wave delineation includes mathematical model based approaches [66,67], signal slope criteria [68,69], second-order derivatives [70], differentiation [71], filtering [72,73], artificial neural networks [74], dynamic time warping [75], statistics [76,77,78] and wavelet transform. Although wavelet methods have been studied in 90-ties [79,80], the most referenced work in this area is the method by Martinez et al. [81]. It uses the information of local maxima, minima and zero crossings at different scales, to identify the QRS complexes and their individual waves (Q, R, S, R’), to delineate the end of T wave and to detect the presence and delineate the borders of P wave. The method further developed by their authors [82] gave birth to a collection of methods exploring phase-free wavelets [83], stationary wavelets [84], or fusion of wavelet and statistical approaches [85].

Studying the state-of-art delineation methods and their possible adaptation to non-uniform ECG representation we identified wavelet-based delineators as the most promising group. Wavelet delineators use time-scale representations, in particular dyadic tree decompositions that correspond to decimated signals [86]. These in turn are particular case of non-uniform representations with sampling interval being a power of two of the basic (i.e., original) discretization time. It is noteworthy that incomplete time-scale representation (i.e., with cut nodes or zeros in leaves of the decomposition tree) may easily be transformed to the temporal domain in time sections corresponding to the gap length with sampling interval multiplied by power-of-two of the empty scale number. However, to implement one of the excellent wavelet delineator of P, QRS and T waves, the inverse transform is needed. Namely, the problem to solve lies in transforming of Non-Uniform signal to time-scale representation (NUTS). Existence of such transform has been confirmed in theoretical works by Liu and Walter [87], Pelissier and Studer [88] and Gröchenig [89]. Zergainoh and Duhamel proved the feasibility of orthogonal decomposition and reconstruction of a signal represented by discrete irregularly spaced samples [90].

The filter-based approach to the wavelet transform [91] or fast wavelet transform cannot be directly applied to non-uniform signals due to uneven spacing of samples. Two approaches were then considered: estimates of spectrum for a non-uniform time series (known as Lomb-Scargle periodogram [92]) and correlation based definition of prototype-based decomposition, as it was originally proposed by Grossman and Morlet [93] and their successors. Unfortunately no spectrum estimate for unequally spaced data is known to have perfect reconstruction property, so with no other option we were to use correlation-based wavelet decomposition. To this point we recall that in the wavelet transform the value of each decomposition coefficient reflects the cross-correlation of the signal and the analyzing wavelet which is a scaled and shifted version of the mother wavelet.

Rehfeld et al. [94] examined three different cross-correlation methods proposed for non-uniform discrete time series: rectangular envelope-based, also called a ‘slotted estimator’ [95] and two weighted estimators with smooth envelopes: Gaussian [96] and Sinc [97]. The slotted estimator uses a simple rectangular time window to slice the observations into discrete sets. In weighting techniques, sudden cutoff of the signal in the time domain is avoided due to the window of smooth shape. All methods have been evaluated with unevenly spaced synthetic signals with various values of time distribution skewness (i.e., ratio of minimum to maximum sampling rate) and scored with the root mean square error (RMSE) to compare their performance. The Gaussian estimator was found the least affected by increasing sampling skewness producing the error of only 2% when the skewness reaches 0.35 (what is typical for non-uniform ECG sampling) and of 12% for the skewness of 2.85. In our case, however, we noticed that the wavelet with compact support is already time-constrained and does not need additional envelope to avoid spectral ripples. This justifies the use of the slotted estimator for its simplicity.

The proposed algorithm of NUTS Transform works in the following two steps:

- Discrete regularly sampled analyzing wavelets $w_{a,b}\left(t\right)$ are located in a time-scale decomposition grid accordingly to their values of scale *a* and shift *b* corresponding to the position of the atom in a pyramid decomposition scheme.
- The correlation coefficient *c* between the non-uniform signal $e\left(t\right)$ and the analyzing wavelet $w_{a,b}\left(t\right)$ is calculated and attributed to the atom at a given position (*a*, *b*) in the grid:
  (17) $$c_{e,w}\left(b,\;s\right)=\frac{\sum_{i=1}^{N^{e}}\sum_{j=1}^{N^{w}}e_{i}w_{j}k_{b}\left(t_{j}^{w}-t_{i}^{e}\right)}{\sum_{i=1}^{N^{e}}\sum_{j=1}^{N^{w}}k_{b}\left(t_{j}^{w}-t_{i}^{e}\right)}$$
  where *e*(*t*) is the electrocardiogram, *w*(*t*) is the analyzing wavelet, *b* is the shift coefficient of the wavelet, *s* is the length of the wavelet support, $k_{b}\left(t_{j}^{w}-t_{i}^{e}\right)$ is the kernel that selects products with time lag not further than half of the length of the wavelet support from its central position:

(18) $$k_{b}\left(t_{i}-t_{j}\right)=\left\{\begin{array}{cc} 1 & \mathrm{for}\;\left|\left|\left(t_{j}-t_{i}\right)\right|-b\right|<\frac{1}{2}\\ 0 & \mathrm{otherwise} \end{array}\right.$$

**Figure 5:**
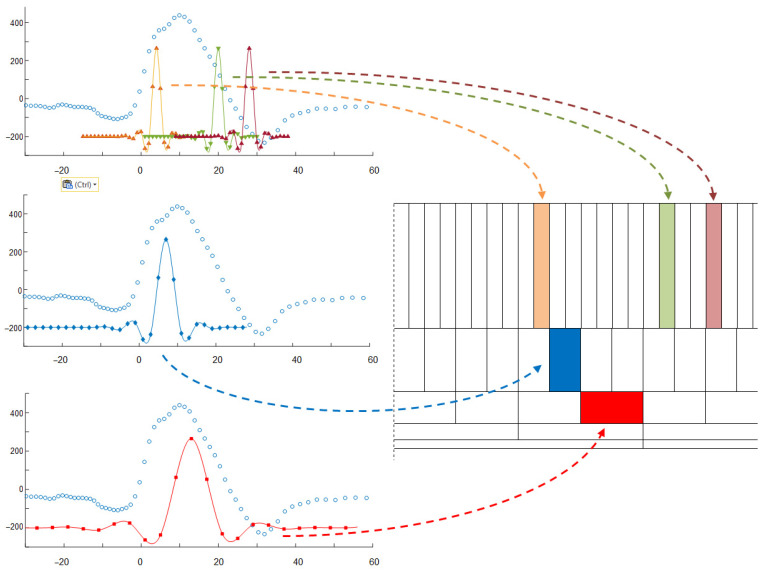
Principle of direct transform of non-uniform signal to uniform time-scale representation.

Iterating the above steps for all the scale *a* and time position *b* values, accordingly to the pyramid decomposition scheme, fills the time-scale grid with coefficients of uniform time-scale transform of a non-uniform time series. Unlike Foster [98] or Cai et al. [99] who discretized the Morlet wavelet to the non-uniform grid, we propose to use reversible wavelet family that allows us for lossless reconstruction of the wavelet signal representation back to the time domain in order to assess the error eventually produced by the proposed direct non-uniform to time-scale transform. Consequently, our choice was the 5-th order Coiflets family [100] being a nearly symmetric, orthogonal wavelet. At each scale *a* the wavelet was shifted in a regular time grid *b* and the cross-correlation *c* with all non-uniform ECG representation points, found in time section corresponding to 30 nonzero coefficients of the wavelet support, was calculated as the ‘slotted estimator’ described above (Figure 5).

Besides the prototype wavelet, originally proposed as quadratic spline [101], we followed the implementation of Martinez algorithm [81] in its wave delineation parts. We considered more sophisticated and little more precise approaches proposed later, but with a goal of demonstration of the non-uniform ECG processing feasibility, we postpone millisecond-based competition between methods to future studies.

**Figure 6:**
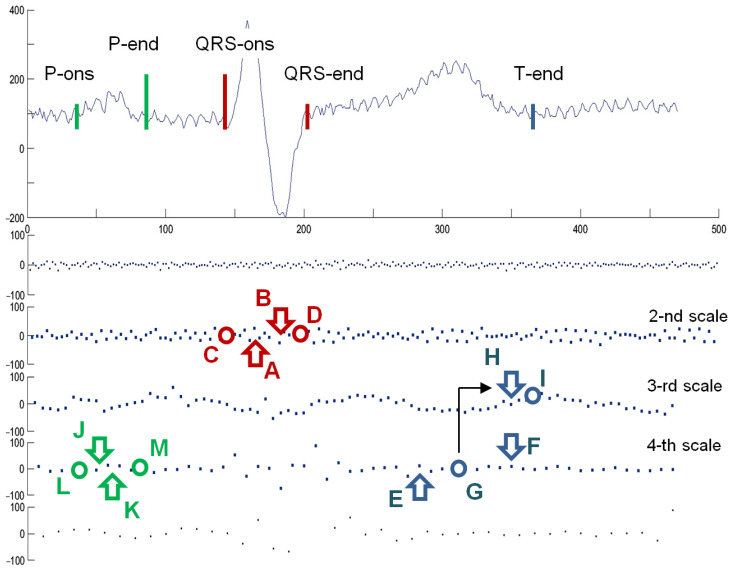
Wave search and delimitation rules in time-scale ECG representation (based on [81], the example is taken from CSE Multilead Database MO_001 lead I). Search points for QRS delimitation points are marked in red, related to T-wave end in blue and related to P-wave borders–in green. Letters A-M refer to explanation of the procedure in the section above.

The delineation algorithm receives the R-wave detection point and first seeks for QRS onset and end by identifying in scale *a* = 2 two consecutive peaks (marked as A and B in Figure 6) of opposite signs associated to the QRS and exceeding a given threshold. Next, local minima are searched before the first peak (C) and after the last peak (D) and also qualified by threshold values relative to peaks’ amplitudes. The T-wave delineation is performed in scales *a* = 3 and *a* = 4 in a time window beginning after the QRS and lasting for a given fraction of local RR interval. In scale *a* = 4 local maxima (E, F) are searched and qualified by another threshold value. Zero crossing (G) between two most significant of them is considered as T-wave peak and to find the T-end, the algorithm returns to scale *a* = 3 and applies similar criteria as for QRS-end (the maximum is marked as H, and the following zero crossing as I). The process is repeated in scale *a* = 5 in case the T-wave was not detected. Finally, the algorithm searches for P-wave in a time window depending on local RR interval preceding the QRS onset point. The search for P-wave is performed in scale *a* = 4 and for qualifying the extremes (J and K) another set of specific thresholds is used. Zero crossing directly preceding the former extreme (L) is considered P-onset. Zero crossing directly following the later extreme (M) is considered P-end. Due to different mother wavelet optimal threshold values were different to those given in Appendix of [81] (Figure 6).

## 5. Test Procedure, Signals, and Conditions

### 5.1. Test Procedure

The principal goal of the paper is to propose basic non-uniform ECG-interpretive procedures and test their applicability to yield diagnostic results possibly equivalent to those obtained from regular software. To this point we applied the commercial interpretive software (MTrace M4Medical, Lublin, Poland) to interpret original test ECGs and their processed counterpart signals and to compare their quantitative and qualitative diagnostic results.

**Figure 7:**
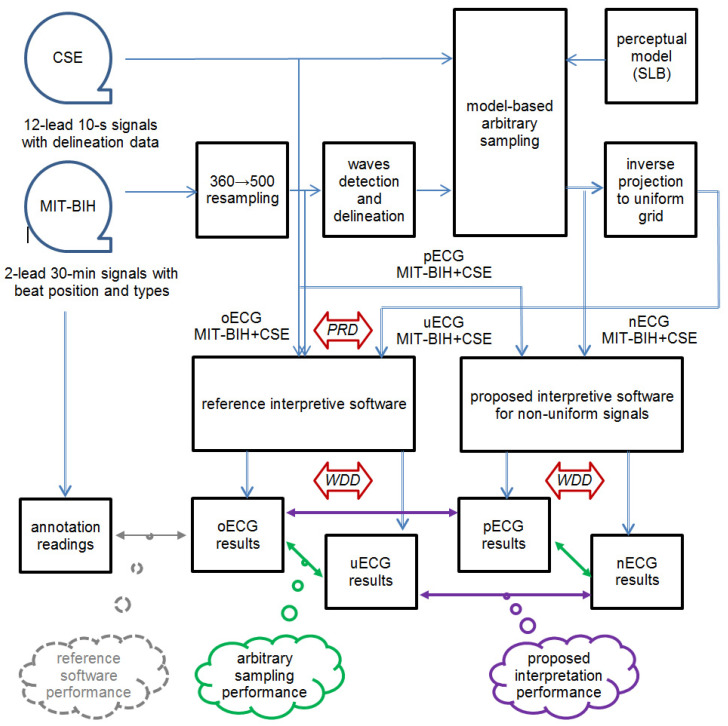
The experiment flow diagram; oECG stands for original records (from both databases), pECG stands for pseudo-non-uniform records (i.e., uniform records in non-uniform data format), nECG stands for non-uniform records (according to arbitrary sampling model) and uECG stands for uniform records produced by projections of nECG to uniform sampling grid.

Two independent processes are possible sources of interpretation errors: (1) arbitrary non-uniform sampling and (2) the interpretation algorithm under test. In the first case, we expect to transform the existing uniform test ECGs (referenced to as ‘original’ or oECG) to the non-uniform sampling grid so as they maintain all original diagnostic information. This may be verified in two ways: either by comparing forward and inverse projected time series (referenced to as ‘uniformized’ or uECG) or by comparing diagnostic results from these series. Assuming that the forward projection of a signal to a sparser sampling grid may be lossy, and the inverse projection may be lossless, we cannot expect bit-identity of oECG and uECG (Figure 7). However, we can expect that the non-uniform representation (referenced to as ‘non-uniform’ or nECG) is not worse (i.e., contains no less information) than the uniformized one (uECG). In other words, we conclude that obtaining identical diagnostic results from corresponding oECG and uECG guarantees that identical diagnostic results are also possible to be calculated from nECG version.

Finally, the proposed interpretive software is expected not to depend on sampling model. Testing all imaginable models would not be practical, but provided the uniformly sampled signal can be considered as a particular case of non-uniform data series we completed the experiment flow by adding a non-uniform analysis of a uniform (i.e., original) ECG record. To this point we only added identical sampling interval information to every sample and called the series of {*t* = 2 ms, *v*} a pseudo-non-uniform signal or pECG. In result we obtain a complete cross-check scheme which consists of:

- reference (i.e., uniform) interpretation of uniform signal oECG,
- uniform interpretation of uniformized signal uECG,
- non-uniform interpretation of non-uniform signal nECG, and
- non-uniform interpretation of pseudo-non-uniform signal pECG.

The experiment flow (Figure 7) was then divided into two parts: first aimed at confirming the coherence of medical information retained in the non-uniform test ECGs and second aimed at evaluating the detector, classifier and delineator procedures proposed in Section 4 to be able to work with non-uniform ECGs. The final comparison, however, goes back to medical result coherence between the original test ECGs and their non-uniform counterparts interpreted with proposed methods. To this point, we separately assess the oECG and uECG signal correspondence and the degree of similarity between results in the domain of diagnostic result.

### 5.2. Error Metrics

Despite of its limitations in signals with variable bandwidth, we estimate the degree of similarity between oECG and uECG signals in the uniformly sampled discrete time domain with the percent root-mean-square difference (PRD) given as:

(19) $$PRD=\sqrt{\frac{\sum_{i=1}^{N}{\left[x_{r}\left(i\right)-x_{s}\left(i\right)\right]}^{2}}{\sum_{i=1}^{N}{\left[x_{r}\left(i\right)\right]}^{2}}}$$

To limit the shortcoming of PRD (and other purely statistical signal metrics, widely used, but loosely related to the medical content of the ECG), besides of calculations of its value in the whole heartbeat, we also provide separate results in the heartbeat’s sections P, QRS and T. Nonetheless, the PRD was only able to compare signals sampled at the same grid, and therefore comparison between oECG or uECG and pECG or nECG required another error measure (WDD, see below) calculated in the domain of quantitative diagnostics parameters.

To assess the performance of the beat detector, common indices as sensitivity (*Se*), positive predictive value (*PPV*), and failed detection (*Fd*) were used. They are defined as (20)–(22):

(20) $$Se=\frac{TP}{TP+FN}\times 100\;\left[\%\right]$$

(21) $$PPV=\frac{TP}{TP+FP}\times 100\;\left[\%\right]$$

(22) $$Fd=\frac{FP+FN}{TP+FP+FN}\times 100\;\left[\%\right]$$

where *TP* (true positive) is the number of correctly detected beats, *FP* (false positive) is the number of detections of non-existing beats, and *FN* (false negative) is the number of undetected existing beats. We consider the detection correct if the time coordinate returned from the detector and the corresponding beat annotation value do not differ by more than 25.6 ms.

To assess the performance of the beat classifier we use confusion matrices and calculate statistics of correctly and erroneously classified beats. Classification statistics has been first computed for 14 most frequent MIT-BIH types (excluding paced ‘P’ and pacemaker-fusion ‘FPN’ beats) and then projected into five AAMI classes of five types (see Section 5.3).

To assess the performance of the wave delineator we follow guidelines of the normative document IEC 60601-2-51 [102] (currently referred to as EN60601-2-25:2015 [103]) specifying required parameters to test, testing conditions, and acceptance threshold values. Three intervals calculated from the wave border points: P wave duration, P-Q interval, QRS complex duration have to show inaccuracy in length of less than 10 ms and the Q-T interval—inaccuracy of less than 30 ms comparing to the mean values of annotation values provided. Amplitude measurement deviations are expected to not exceed ±25 µV (or 5% for amplitudes above 500 µV).

To assess the coherence of diagnostic results we applied Weighted Diagnostic Distortion (WDD) [104]—a comprehensive error metric based on diagnostic features. It is based on comparing the PQRST complex features of the two ECG signals and was defined as:

(23) $$WDD\left(\beta ,\hat{\beta }\right)=\Delta \beta^{T}\frac{\Lambda }{tr\left(\Lambda \right)}\Delta \beta \cdot 100$$

where ∆*β* is the normalized difference vector between original and processed PQRST features, *β* and $\hat{\beta }$ represent two vectors of 18 diagnostic features (RR_int._, QRS_dur._, QT_int._, QTp_int._, P_dur._, PR_int._, QRS_peaks_no._, Q_wave_exsist._, Δ_wave_exsist._, T_shape_, P_shape_, ST_shape_, QRS(+)_amp._, QRS(-)_amp._, P_amp._, T_amp._, ST_elevation_, ST_slope_) of compared beats, and ∧ is a diagonal matrix of weights heuristically set to [105]:

∧ = diag [2.5 2.5 1 1 2 2 1 0.5 0.1 1.5 1 3 1.5 1.5 1 1 3 3](24)

The WDD is ECG-specific error metric providing results related to medical findings equivalence rather than to signal representation accuracy. Therefore it is more adequate to evaluate the preservation of medical content in signals with irregular distribution of information and, thanks to sampling-independence, also can estimate the equivalence between interpretation results by commercial software and proposed procedures despite the latter work with non-uniform ECGs. The only drawback of this metric is, however, the necessity of using interpretive software, which, if originated from different manufacturers, may affect the repeatability of results.

The verbose diagnosis is an automatically generated text message inferred from quantitative results of the ECG interpretation accordingly to worldwide standardized rules [4]. It is meant to suggest possible diagnosis to a human cardiologist, who confirms or corrects the findings. The message is composed of a phenomena descriptor (such as: ‘Sinus tachycardia’ or ‘Supraventricular rhythm’) and an intensity- or occurrence-related modifier (such as: ‘moderate’, ‘persistent’, ‘occasional’ etc.). The collection of descriptors and modifiers forms a closed list and is represented by labels in the interpretive software code. We used statistics of labels matching as a qualitative measure of changes caused by non-uniform ECG representation and processing.

### 5.3. Test Signals

We used test ECG signals from two databases commonly used for testing of commercial ECG software. The MIT-BIH Arrhythmia Database (MITDB) [5], was published in late 1970s and since then it has been a worldwide standard for testing long term ECG recorder interpretive software. The database contains 48 two-leads half-hour records sampled at 360 Hz (i.e., sampling interval td = 2.778 ms) with a resolution of 11 bits over a ±5 mV range. Since paced ‘P’ and pacemaker-fusion ‘FPN’ beats are not compatible with the arbitrary sampling model, records 102, 104, 107 and 217 were excluded from the study with all beats of other types they contain. Except for rare cases of noisy signals, we used both available leads, however, for the reason of compatibility, the records were resampled to 500 Hz using cubic splines. The MITDB Database provides annotations with heartbeat positioning and type, therefore, as many other researchers, we used it to evaluate both the QRS detection and the beat classification procedures. The original information of beat positions was multiplied by 500/360 due to signal resampling and the original 41 beat types has been first reduced to 14 most frequent (at least 16 cases, ‘P’ and ‘FPN’ excluded) and then projected into five-classes: Normal, supraventricular, ventricular, fusion and unknown according to the ANSI/AAMI EC57:1998 standard [106] (Table 1).

The CSE database inherited its name from the project ‘Common Standards for quantitative Electrocardiography’. It was completed in 1989 and targeted at quantitative standardization of ECG measurement procedures [6]. The database is a worldwide reference set of multilead electrocardiographic records (500 Hz, 0.25 µV resolution), accompanied by their basic measurement results. Besides 125 15-lead 10 s records, it provides information of the reference beat location and all wave border positions: P-onset, P-end, QRS-onset, QRS-end and T-end calculated by 19 automatic interpretation software sets. As the results show beat-to-beat variability, testing the wave delineation accuracy is not possible with original files (i.e., ‘MO’ dataset) [71]. Instead, we used the corresponding ‘MA’ dataset that reproduces the original reference beat for 10 s making all beats identical and the wave delineation results-independent of beat selection. Similarly to the MITDB Database, we excluded CSE records 67 and 70 due to containing paced beats.

Both groups of test signals have been further processed to produce arbitrarily sampled test ECGs accordingly to the proposed sampling model. In general, the non-uniform sampling assumes each sample to be a pair of time and amplitude values. We adhere to this assumption throughout this paper, and all tests of proposed interpretive software for non-uniform ECG were performed with series of {*t*, *v*} pairs where *t* is monotonic continuous time coordinate represented in floating point data type (indeed, the sample may be taken anytime in given range of sampling interval 2–10 ms) and *v* is quantized value of the signal represented in 16-bit integer data type. Optimization of time representation in non-uniform signals, although very welcome in practical implementation, falls beyond the scope of this paper.

### 5.4. Perceptual Information Distribution as Arbitrary Sampling Model of the ECG

In arbitrary non-uniform sampling the values are not equidistant and the sampling interval information is stored along with the value series. In a general case samples may fall anywhere, provided the values of sampling time are strictly monotonic. However in quasi-periodic variable-bandwidth signal the usage of a sampling model successively adapted to inter-period variations of the signal is by far more efficient. The sampling model is adequate if it reflects local bandwidth variability of the signal (and thus called ‘standardized local bandwidth’, SLB). The model should also be anchored to a certain fiducial point of each period of the signal.

Several approaches to non-uniform sampling of the ECG have been presented such as compressed sensing [107,108], mutual data dependence [109,110] or level-crossing sampling [111,112], However in the case of ECG, a reliable expectation of local bandwidth in each heartbeat (i.e., sampling model) has to consider the electrophysiology of the heart. The ECG recorded from given points on the skin surface represents a complex process of generation and conduction of the stimulus through the heart tissue. Respectively, the action of atria is represented in P-wave, the action of ventricles—in QRS complex and the subsequent T-wave stands for repolarization of cells in ventricles. This justifies the medical importance of wave borders delineation, since the borders separate the elementary actions in the heart cycle and are used for calculation of intervals in their sequence such as PQ or QT. Since the electrophysiological properties and thus the conduction conditions differ between the atria and ventricles tissue and between the depolarization and repolarization processes, wave borders are also informative about bandwidth variations that can be expected in progression of the cardiac cycle. Studies of variability of the intervals in the cardiac cycle show that although in general all of them shrink when the cardiac cycle shortens (and vice-versa), the local shortening is not proportional and therefore all wave borders have to be considered to locally squeeze or stretch the SLB. In this way, for every heartbeat present in test ECG records, the wave borders were determined (or read from the database annotation record) and used for individual adaptation of the SLB to the time-course of that beat that leads to a series of sampling intervals.

As mentioned above, the expected local bandwidth of the signal may reasonably be justified by the physiology. In our previous experiments we already tried to estimate the generalized medical relevance function for the ECG from:

- local bandwidth of ECG waves [113],
- susceptibility of diagnostic result to signal distortion caused by a local data loss, and
- local conspicuity of the ECG trace [114,115].

The latter method has been further developed as a background for adaptive sampling and recently published [116]. The SLB defining the arbitrary sampling model applied in our experiments is directly calculated from the generalized medical relevance function proposed in that paper.

### 5.5. Processing of Test Signals Accordingly to the Arbitrary Sampling Model

We studied various approaches to make the arbitrary sampling model usable for heartbeats with P, PQ, QRS and QT sections of length varying in wide range. Initially, a piecewise linear projection of the model to actual wave border positions was proposed [116] for its computational simplicity. Distortions eventually caused by stepwise changes of model sampling (i.e., squeezing or stretching of the generalized medical relevance function) were not noticed. Later we developed more sophisticated scaling transform [117] that projects general SLB pattern to the actual strip of signal in three steps: (1) calculation of resampling factor to compensate for different length of corresponding sections in ongoing ECG signal and in generalized medical relevance function, (2) extrapolation of local resampling factor values to a discrete time function and interpolation with cubic splines with nodes falling exactly in sections’ borders and (3) resampling of the SLB function with splines interpolation according to the local resampling value.

**Figure 8:**
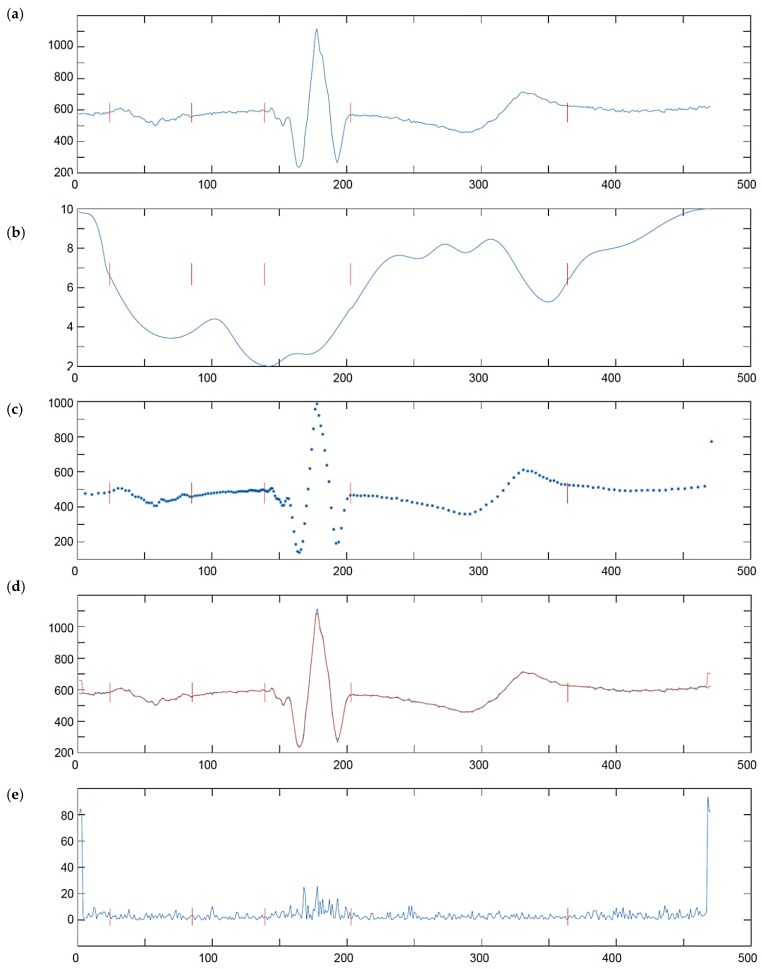
CSE test file MA1_001, lead I (**a**) oECG, (**b**) sampling interval [ms], (**c**) nECG, (dot-plotted to highlight non-uniform sampling), (**d**) uECG (orange) vs. oECG (blue), (**e**) absolute uniformization error (|oECG-uECG|); all horizontal axes in sample numbers (a 2 ms unit); vertical red lines are P, QRS and T-waves delimitation points; errors at both ends of nECG and uECG are caused by adaptive anti-aliasing filter.

**Figure 9:**
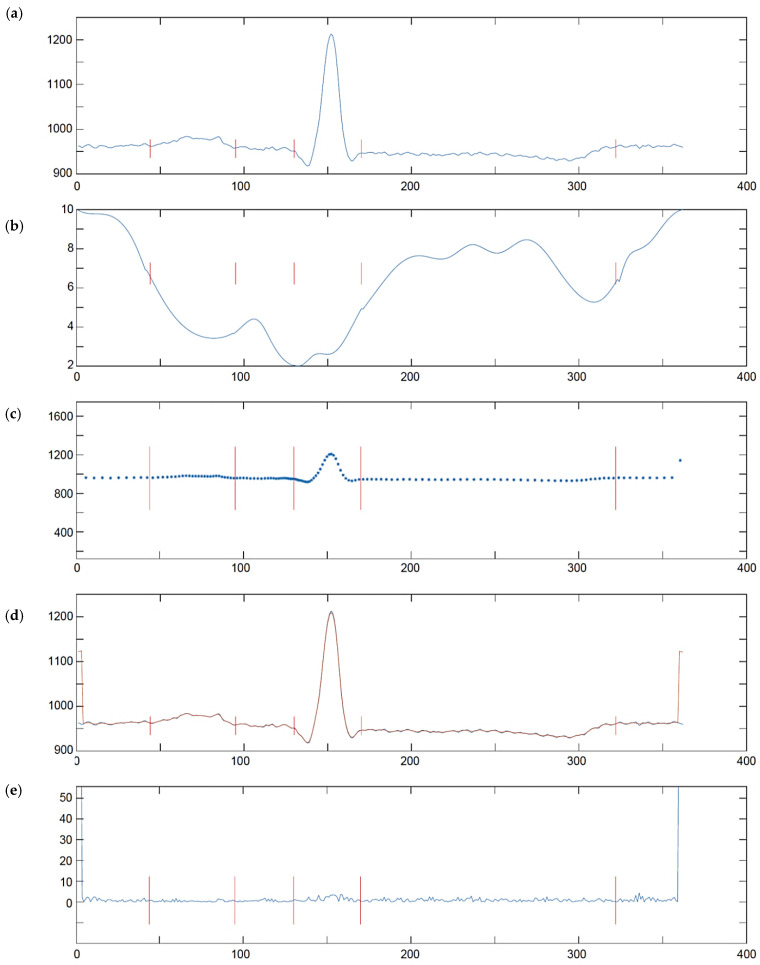
MITDB Database test record 100, lead II (**a**) oECG, (**b**) sampling interval [ms], (**c**) nECG, (dot-plotted to highlight non-uniform sampling), (**d**) uECG (orange) vs. oECG (blue), (**e**) absolute uniformization error (|oECG-uECG|); all horizontal axes in sample numbers (a 2 ms unit); vertical red lines are P, QRS and T-waves delimitation points; errors at both ends of nECG and uECG are caused by adaptive anti-aliasing filter.

Transforming of MITDB and CSE database signals to their arbitrarily sampled version is based on wave delimitation points. In case of CSE Multilead Database (Figure 8), their positions are provided in the annotation file and were read directly. In case of MITDB Database (Figure 9) the annotation file contains only detection and beat type references. Therefore, we first processed the reference files with a single lead wave delineator (by Aspel, Zabierzów, Poland) and then applied arbitrary sampling according to the wave borders detected. To avoid delineation errors, we applied the arbitrary sampling mode to the 14 most frequent heartbeat types as annotated in the reference file accompanying each MITDB record. The remaining (i.e., rare) heartbeat types have been left untouched i.e., used the sampling rate of 500 Hz.

## 6. Test Results

### 6.1. Results of QRS Detection Test

The detection performed by the proposed interpretive software was tested with MITDB test files in both nECG and pECG variants. The detailed file-by-file results for nECGs expressed in *Se*, *PPV* and *Fd* ratios are presented in Table 2.

Corresponding results achieved with pECG MITDB records were the following: *TP* = 100,791, *FP* = 100, *FN* = 75, *Se* = 99.9337%, *PPV* = 99.8966%, and *Fd* = 0.1695%.

Comparison of the overall detection performance between the state-of-art uniform detectors and the proposed non-uniform detector is presented in Table 3.

### 6.2. Results of QRS/Beat Classification Test

The performance of proposed heartbeat classification method was evaluated with MITDB test files in both nECG and pECG variants, first against 14 MITDB beat labels and then against the ANSI/AAMI EC57:1998 standard beat labels. Table 4 presents the confusion matrix for all 14 beat types.

In nECG records from MITDB clustered according to MITDB beat labels the total number of misclassified beats (out of the diagonal of confusion matrix) was 740 out of the total of 101,352, what means the classification correctness of 99.27%. Corresponding values for pECG records were: 731 and 99.28% respectively.

Table 5 displays the confusion matrix for the ANSI/AAMI EC57:1998 standard beat labels.

When the same nECG records from MITDB were clustered according AAMI categories the total number of misclassified beats (out of the diagonal of confusion matrix) was 637, what means the classification correctness of 99.37%. Corresponding values for pECG records were: 632 and 99.38%, respectively. The score is higher than in case of MITDB categories, because misclassified MITDB types falling under the same AAMI label are no longer considered as misclassified.

### 6.3. Results of QRS Waves Delineator Test

The wave delineator has been tested with 123 CSE Multilead database signals from the ‘MA’ set (files 67 and 70 with pacemaker beats were excluded) in both nECG and pECG variants. This set consists of representative beat taken from corresponding ‘MO’ (i.e., Original) record and repeated until filling the 10 s signal strip.

The CSE Database comes with delineation results provided by 19 interpretive software packages participating in the CSE project [6]. In each file the results for each fiducial point are sorted in ascending order precluding disclosure of particular package results, but authorizing calculations of:

- Average position of the fiducial point, being an approximate of the ground truth.
- Standard deviation of the results, being an approximate of difficulty level in getting a consistent result; it is noteworthy that due to various medical content and recording conditions, particular records pose different challenges to the interpretive software making the results more or less reliable; consequently, absolute values of position difference cannot be directly collected from all files.
- Rank of the delineator under test given accordingly to ascending absolute difference of calculated or provided and ground truth result; the rank *R* displayed in Table 6 for four proposed testing scenarios was calculated among 19 reference interpretive software from CSE database.

In our experiment we used oECG and uECG with the reference interpretive software and nECG and pECG with the proposed interpretive software to obtain four sets of results of which none was the ground truth:

- Original signal (oECG) results, in which the delineation accuracy was affected by limited precision of reference interpretive software,
- Uniformized signal (uECG) results, in which the delineation accuracy was affected by limited precision of reference interpretive software and non-uniform representation of the signal (however the use of reference software required transforming the nECG back to the uniform sampling space),
- Non-uniform signal (nECG) results, in which the delineation accuracy was affected by limited precision of proposed delineator under test and non-uniform representation of the signal.
- Pseudo-non-uniform signal (pECG) results, in which the delineation accuracy was affected only by limited precision of proposed delineator under test.

Table 6 summarizes the differences ∆Time (∆T) and the Rank (R) of each result set (each of 123 files and 5 fiducial points) against the ground truth data read from CSE reference file.

The ∆Time is the time difference between the calculated and the reference positions of wave border. Positive values mean too late delineation, whereas negative values stand for too early. In the uniform space (i.e., in oECG and uECG) the delineator always points at integer sample number, and since the reference is real-valued the ∆Time results repeat the fraction part of the average result. In non-uniform space (i.e., in nECG and pECG) the wave border position may fall between samples and must be expressed in real numbers.

The delineation accuracy summary in a form complying with IEC EN60601-2-25:2015 [103] is presented in Table 7.

Besides the error in wave border delineation, the experiment allows us to assess the arbitrary sampling and non-uniform ECG interpretation algorithm in two complementary error metrics: the most commonly used PRD (measuring the identity of digital signals) and the WDD (measuring the identity of diagnostics results, see Section 5.2). The result of this assessment is presented in Table 8.

Finally, we check and compare how far the labels of diagnostic statements [4] proposed by the interpretive software matches for original, uniformized, non-uniform, and pseudo-non-uniform records from CSE Database. The result of this comparison is presented in Table 9.

In Table 9 particular columns of results concern four testing scenarios. In case of oECG the results were inaccurate due to the interpretive software embedded in the electrocardiograph used. In case of uECG, the results have been additionally affected by the non-uniform representation although after re-uniformization they were interpreted by the same software. The difference between results for uECG and oECG shows the extent of affecting the medical content with non-uniform representation of the signal measured by reference software; while difference between results for uECG and oECG does the same by the proposed software (see Figure 7 green arrows). These results vary accordingly to the arbitrary sampling model used. In case of nECG the results have been additionally affected by the proposed delineation software, developed to process non-uniform ECGs (see Section 4.3). In case of pECG the proposed delineation software processed the uniform signal, so the influence from to the arbitrary sampling model was removed. Consequently, the difference between nECG and uECG results is a measure of quality of this software with use of non-uniform signal; while the difference between pECG and nECG results does the same with use of uniform signal (see Figure 7 violet arrows).

## 7. Discussion

The main novelty of this paper and the principal achievement of the reported research is the development and testing of a complete ECG interpretation chain designed for processing non-uniform signals. The question about feasibility of medical diagnostics based on arbitrary non-uniform ECG representation has found a positive answer. To this point a QRS detection method, a beat classification method and wave delineator, all three adapted to irregular sampling, have been proposed and tested accordingly to industrial standard used in evaluation of medical-grade equipment.

In designing of new algorithms all state-of-art solutions were considered and evaluated as candidates to work with non-uniform time series. The latter criterion eliminated several high quality procedures e.g., those assuming digital filtering or transforms.

In testing phase we did our best to separate the data inconsistency originated from the arbitrary sampling model and inaccuracy caused by imperfect interpretation procedures. All this have to be measured with real interpretive software, none of which is expected to provide ground-truth result. To this point we used: (1) original database signals (referred to as oECGs), (2) their non-uniform counterparts (nECGs) calculated with arbitrary sampling model, and a cross-validation scheme based on two complementary signal sets: (3) uECGs being uniformized (i.e., projected to uniform sampling grid) nECG signals, and (4) pseudo-non-uniform (pECG) records being oECG signals stored in the {time, value} format suitable for non-uniform processing.

Comparison of results between oECG and uECG (Table 8 column 5) reveals differences between uniform and non-uniform ECG content in uniform processing domain (the reference interpretive software was used), while comparison of results between pECG and nECG (Table 8 column 6) does the same in non-uniform processing domain (the proposed interpretive software was used). In both cases the results were attributed to the correctness of choice of the arbitrary sampling model. The perceptual wave border-dependent model with sampling rate variation between 100 and 500 Hz with only mild compression of the ECG (i.e., average compression ratio of 3.1 [116]) was found adequate to not alter the WDD by more than 0.23% (worst case), to preserve the correctness of 115 out of 121 (i.e., 95.0%, worst case) final statements and to maintain quantitative results inaccuracy within the tolerance margin allowed by IEC normative document.

The difference of results between uECG and nECG (Table 8 column 7) shows how accurate were the proposed (i.e., non-uniform signal-adapted) interpretive procedures working with non-uniform ECG signals. Similarly, the difference of results between oECG and pECG (Table 8 column 8) shows how accurate were the proposed interpretive procedures working with uniform ECGs. In this case mismatch of verbose statement was found only in 4 out of 121 cases (i.e., 3.3%, worst case), however the WDD value was slightly higher (0.33% worst case) and also quantitative errors of wave delineation were higher, but still allowable for medical-grade instruments. It is noteworthy that the result of comparison is meaningful in relation to the reference software (i.e., the absolute truth is not known).

Finally the difference of results between nECG and oECG (Table 8 column 9) cumulates the diagnostic results inconsistency due to the arbitrary sampling model in use and inaccuracy caused by imperfect interpretation procedures. As expected, the value of cumulative error is higher than values of each error produced by the model and by the procedure, but alteration of the quantitative diagnostic results does not exceed 0.5% what seems to be of marginal significance in medical aspect. It is also noteworthy, that the commonly used PRD metrics was only adequate to technically compare signals of mutually corresponding samples (i.e., between uECG and oECG), while the WDD, although more complex to calculate, was applicable to comparisons of data from medical viewpoint not regarding their background signals sampling rate.

The paper also opens new area of research focused on optimization of each subsequent step of ECG interpretation. These investigations may be targeted either to better accuracy or to lower computational complexity of the procedure. Despite disparate results of comparison to the state-of-art procedures, varying from ‘excellent’ for beat detection to ‘average’ for wave delineation, the processing chain in the whole fulfills the industrial standard [102] for Essential Performance of Electrocardiographs and can be considered as embedded part of medical device or as a standalone Software as Medical Device (according to current Medical Device Regulations). Consequently, main research effort should be directed to propose less computationally demanding procedures of comparable outcome quality. To this point evaluation is to be done in parallel in the diagnostic quality and in computational complexity metrics.

In the proposed heartbeat detector, subsequent values of *t* (Equations (5) and (6)) are independent of the window length Δ*t* what allows us to calculate the DF values at arbitrarily given points. For the same, one may use a dense grid (with overlapping windows) when high precision is required or sparse grid (with disjoint windows) to increase processing speed. In case of multimodal signal employing independent arbitrary sampling models in each of its components, the DF may be calculated commonly. From the viewpoint of hardware implementations, particularly in mobile heart rate monitors, an important advantage of the detection algorithm is computational simplicity. The only computation-demanding operation seems to be the arctangent (Equation (8)); however, an approximation of desired precision may be obtained by truncating infinite series calculated according to [26]:

(25) $$\tan^{-1}(z)=\sum_{n=1}^{\infty }\frac{{\left(-1\right)}^{n}\cdot z^{2n+1}}{2n+1}$$

Studying the behavior of the beat detector we noticed very rare detections closer than 200 ms of each other. Such double detections are problematic for most state-of-art methods and several measures were proposed in the literature (such as ‘eyeclosing’ in Pan-Tompkins paper [21]), but the proposed detector does not seem to require one. The most plausible reason is the smoothing effect of the robust regression (Nadaraya-Watson) applied, the legs of long time triangle *w_L−_*, *w_L_* span for 122 ms. Moreover, multiplication of angles between long time triangle legs and concurrently between short time triangle legs leads to unambiguous identification of significant turn of electrical activity of the heart referred to as heartbeat peak

In further investigations of the heartbeat detector one may explore potential benefit from the information on local sampling rate which originates from arbitrary sampling model and is expected to show beat-to-beat repeatability at least in normal rhythm.

In beat classification algorithm the only constraint implied by non-uniform sampling lies in complicated calculation of the objects’ (beats’) distance (Equations (15) and (16)). The proposed classifier has good performance expressed by the classification accuracy of 99.27 (MITDB) or even 99.37 (AAMI). These figures well correspond to results of best known QRS classifiers, however it’s not surprising because in case of aberrant patterns when initial delineation was problematic, the sampling model used uniform sampling of maximal rate (500 Hz). Despite the statistics, separation of some patterns rarely represented in MIT-BIH Database (e.g., Nodal Premature Beats—J, Escape Beats ‘e’ or ‘E’) is still not satisfactory. Usage of high definition class kernels, proposed here for non-uniform patterns but not used in state-of-art methods, also contributed to relatively good result of our method by reducing pattern-to-kernel misalignment factor. Considering the total number of heartbeats in a longitudinal ECG record, the calculation of distance between non-uniformly sampled signals is computationally very expensive and its improvement may be a challenge for further research. Signal registration, dynamic time warping, mutual information or likelihood maximization are first hand techniques to apply and evaluate.

Wave delineation algorithm has been proposed based on a direct transform of non-uniformly sampled signal to the time-scale representation (NUTS). The innovation behind consists in making a step back to the correlation-based definition of wavelet transform and calculating the cross-correlation between signal samples and the wavelet at the arbitrary sampling points (Equation (17)). Additional envelope, regularly used to avoid spectral ripples, was not necessary due to decaying slopes of the wavelet. Despite the non-uniform sampling, the resulting time-scale representation uses a regular dyadic decomposition grid and the remaining part of the delineation algorithm may perform as proposed by Martinez [81]. Indeed, the delineation accuracy results for nECG and pECG are worse than for original and uniformized signals (i.e., oECG and uECG respectively); however still satisfy requirements given by the industrial standard. This finding is also supported by negligible mismatch of diagnostic statements issued by the reference interpretive software and the software under test based on wave delineation and amplitude measurements. In wave delineation task the NUTS transform, originated from correlation-based wavelet decomposition, was found a primary source of computational load. In this aspect finite collection of deformable heartbeat patterns and non-uniform node interpolation technique are expected to yield a computationally effective and accurate solution.

As it was demonstrated in this paper, the use of perception-based arbitrary sampling does not alter diagnostic meaning of the ECG signal (in terms of IEC regulations). Tests have been performed with a general-purpose database to give broad aspect of performance of proposed method. Nevertheless other sampling models are equally applicable according to particular needs for reproducing selected parts of the ECG more or less accurately. Studies on records from patients with confirmed particular diseases will lead to optimization of sampling models appropriately to the a priori knowledge about the patient (following the paradigm of personalized medicine). In this aspect, interpretive methods presented in this paper show another advantage: independence of the sampling model partly proven with pseudo-non-uniform records. Although the quality of result may vary with the model and disease match, the proposed processing scheme works correctly for all ECG signals with sampling in given frequency range. Consequently, the presented method is universal, sampling-independent and ready to use with virtually any sampling model including the flat one (i.e., constant sampling frequency), we commonly use today.

Although better results can still be obtained with first uniformizing the non-uniform ECG representation and then interpreting it as a uniform ECG by regular interpretive software, we demonstrated the feasibility of purely non-uniform interpretation of ECG, measured the loss of accuracy separately caused by arbitrary sampling model and by non-uniform signal processing and pointed out possible ways of further improvement in algorithms for non-uniform ECG interpretation.

## Figures and Tables

**Table 1:** MITDB labels of the heartbeats and corresponding standard AAMI classes.

AAMI Type	AAMI Label	MITDB Type and Label	Total
Normal	N	Normal (NOR)—N Left Bundle Branch Block (LBBB)—L Right Bundle Branch Block (RBBB)—R	89,838
Supraventricular ectopic	S	Atrial Premature Contraction (APC)—A Nodal (Junctional Escape Beat)—j Blocked Atrial Premature Beat (BAP)—x Aberrant Atrial Premature Beat (AP)—a Nodal (Junctional) Premature Beat (NP)—J Atrial Escape Beat (AE)—e	3217
Ventricular ectopic	V	Premature Ventricular Contraction (PVC)—V Ventricular Flutter (VF)—! Ventricular Escape Beat (VE)—E	7480
Fusion	F	Fusion of Ventricular and Normal Beat (VFN)—F	802
Unknown	Q	Unclassificable Beat (UN)—Q	15

**Table 2:** Summary of performance ratios for individual nECG MITDB files.

MITDB File ID	Results						
	Beats	TP	FP	FN	Se [%]	PPV [%]	Fd [%]
100	2273	2272	0	1	99.96	100.00	0.04
101	1865	1864	3	1	99.95	99.84	0.21
103	2084	2083	0	1	99.95	100.00	0.05
105	2572	2568	13	4	99.84	99.50	0.66
106	2027	2026	0	1	99.95	100.00	0.05
108	1774	1773	28	1	99.94	98.45	1.61
109	2532	2532	0	0	100.00	100.00	0.00
111	2124	2124	1	0	100.00	99.95	0.05
112	2539	2539	0	0	100.00	100.00	0.00
113	1795	1795	0	0	100.00	100.00	0.00
114	1873	1871	9	2	99.89	99.52	0.58
115	1953	1953	0	0	100.00	100.00	0.00
116	2412	2396	1	16	99.34	99.96	0.70
117	1535	1535	0	0	100.00	100.00	0.00
118	2288	2288	1	0	100.00	99.96	0.04
119	1987	1987	1	0	100.00	99.95	0.05
121	1863	1862	0	1	99.95	100.00	0.05
122	2476	2476	0	0	100.00	100.00	0.00
123	1518	1518	0	0	100.00	100.00	0.00
124	1619	1619	0	0	100.00	100.00	0.00
200	2601	2600	1	1	99.96	99.96	0.08
201	2000	1998	0	2	99.90	100.00	0.10
202	2136	2136	1	0	100.00	99.95	0.05
203	2980	2969	16	11	99.63	99.46	0.90
205	2656	2655	0	1	99.96	100.00	0.04
207	2332	2332	3	0	100.00	99.87	0.13
208	2955	2946	1	9	99.70	99.97	0.34
209	3005	3005	1	0	100.00	99.97	0.03
210	2650	2633	3	17	99.36	99.89	0.75
212	1825	1825	1	0	100.00	99.95	0.05
213	3251	3250	0	1	99.97	100.00	0.03
214	2262	2261	0	1	99.96	100.00	0.04
215	3363	3360	0	3	99.91	100.00	0.09
219	2287	2287	0	0	100.00	100.00	0.00
220	2048	2048	0	0	100.00	100.00	0.00
221	2427	2426	0	1	99.96	100.00	0.04
222	2483	2483	3	0	100.00	99.88	0.12
223	2605	2604	0	1	99.96	100.00	0.04
228	2053	2452	13	1	99.96	99.47	0.57
230	2256	2256	0	0	100.00	100.00	0.00
231	1573	1573	0	0	100.00	100.00	0.00
232	1780	1780	3	0	100.00	99.83	0.17
233	3079	3078	0	1	99.97	100.00	0.03
234	2753	2753	1	0	100.00	99.96	0.04
Sum:	100,469	100,791	104	78			
Average:					99.9309	99.8928	0.1761

**Table 3:** Performance of the proposed algorithm with nECG records compared to selected other work.

Algorithm	Results						
	Total	TP	FN	FP	Se [%]	PPV [%]	Fd [%]
Pan and Tompkins (1985) [21]	109,809	109,532	277	507	99.75	99.54	0.71
Martínez et al. (2004) [81]	109,428	109,208	220	153	99.80	99.86	0.34
Yeh and Wang (2008) [118]	109,809	109,643	166	58	99,85	99,95	0.20
Martínez et al. (2010) [24]	109,428	109,111	317	35	99.71	99.97	0.32
Zidelmal et al. (2012) [119]	109,494	109,101	393	193	99,64	99,82	0,54
Song et al. (2015) [26]	109,494	109,398	96	103	99.91	99.91	0.18
proposed algorithm *	100,469	100,791	104	78	99.93	99.89	0.18

* Results displayed in Table 2 bottom line are here rounded to two decimal points for data consistency.

**Table 4:** Confusion matrix for beat classification using MITDB beat labels (with nECG MITDB records, labels concatenated accordingly to AAMI have the same background).

Ref. Class	Determined Class														Total *
	N	L	R	A	j	x	a	J	e	V	!	E	F	Q	
**N**	73,980	32	0	253	37	7	35	0	13	73	27	0	47	7	74,511
**L**	12	8053	0	0	0	0	0	0	0	7	0	0	0	0	8072
**R**	27	0	7197	15	0	0	9	3		4	0	0	0	0	7255
**A**	5	5	0	2518	0	0	0	0	0	5	3	0	10	0	2546
**j**	4	0	0	5	217	0	0	3	0	0	0	0	0	0	229
**x**	7	0	0	2	1	180	0	0	0	0	3	0	0	0	193
**a**	4	0	1	1	0	0	144	0	0	0	0	0	0	0	150
**J**	2	0	0	2	0	0	0	79	0	0	0	0	0	0	83
**e**	3	1	0	0	0	0	0	0	12	0	0	0	0	0	16
**V**	11	5	0	0	0	0	0	0	0	6878	7	0	1	0	6902
**!**	0	8	0	0	0	0	0	0	0	11	453	0	0	0	472
**E**	5	0	0	0	0	0	0	0	0	0	0	101	0	0	106
**F**	7	0	0	0	0	0	0	0	0	2	0	1	792	0	802
**Q**	2	1	0	0	0	0	0	0	0	3	0	1	0	8	15
**Total**	74,069	8105	7198	2796	255	187	188	85	25	6983	493	103	850	15	101,352

* MITDB records 102, 104, 107 and 217 with paced ‘P’ and pacemaker-fusion ‘FPN’ beats were excluded.

**Table 5:** Confusion matrix for beat classification using AAMI beat labels (with nECG MITDB records).

Reference	Determined Beat Type					Total
	N	S	V	F	Q	
**N**	89,301	372	111	47	7	89,838
**S**	32	3164	11	10	0	3217
**V**	29	0	7450	1	0	7480
**F**	7	0	3	792	0	802
**Q**	3	0	4	0	8	15
**Total**	89,372	3536	7579	850	15	101,352

**Table 6:** Excerpt from the detailed analysis of wave delineation result with CSE database (files 67 and 70 are excluded).

CSE NR	Fiducial Point	CSE-Reference		oECG		uECG		nECG		pECG	
		Mean	std.	∆T	R	∆T	R	∆T	R	∆T	R
001	P-ons	51.26	13.99	4.26	3	5.26	4	5.71	5	4.51	3
	P-end	170.6	19.98	−3.63	3	-3.63	3	−2.28	2	−2.61	2
	QRS-ons	277.6	5.57	1.58	2	2.58	5	2.77	6	1.91	3
	QRS-end	407.6	4.48	1.58	3	2.58	6	2.80	6	1.88	4
	T-end	723.5	12.50	3.47	3	4.47	5	5.11	6	3.85	3
002	P-ons	20.89	9.34	3.89	4	3.89	4	4.41	5	4.06	4
	P-end	135.8	7.51	−2.78	3	−2.78	3	−4.11	5	−3.50	4
	QRS-ons	178.2	2.82	0.21	1	1.21	6	1.57	8	0.59	2
	QRS-end	264.1	5.33	−1.11	2	−1.11	2	−1.93	5	−1.31	2
	T-end	523.1	54.70	7.11	4	8.11	4	8.51	4	7.91	4
			…120 files…								
125	P-ons	96.63	6.62	2.62	4	2.62	4	2.97	5	2.91	5
	P-end	186.0	4.86	2.0	3	2.0	3	2.57	5	2.13	3
	QRS-ons	206.8	2.31	0.84	2	0.84	2	1.12	3	0.99	3
	QRS-end	314,2	4.53	−1.21	3	−1.21	3	−1.85	5	−1.02	2
	T-end	612.6	6.56	1.63	3	2.63	5	2.90	5	2.83	5
Average rank (out of 19 packages):					2.87		3.93		5.0		3.37

**Table 7:** Accuracy of waves and sections delineation in original, uniformized, non-uniform, and pseudo-non-uniform ECG [ms].

ECG Section	oECG		uECG		nECG		pECG		IEC Allowed Tolerance	
	Mean	std.	Mean	std.	Mean	std.	Mean	std.	Mean	std.
**P**	±4.3	3.5	±7.7	6.8	±9.7	8.1	±5.7	4.1	±10	15
**QRS**	±2.1	1.8	±2.8	3.7	±3.4	5.1	±2.8	3.2	±10	10
**P-Q**	±3.7	4.5	±5.4	6.7	±6.7	8.4	±4.1	4.9	±10	10
**Q-T**	±11.1	7.1	±12.8	8.8	±17.7	10.9	±11.7	8.1	±25	30

**Table 8:** Signal error (PRD) and diagnostic result error (WDD) measures (CSE database, files 67 and 70 are excluded); all values in [%].

CSE-NR	uECG—oECG				WDD				
	PRD	PRD P	PRD QRS	PRD T	oECG- uECG	pECG- nECG	uECG- nECG	oECG- pECG	nECG- oECG
Column	1	2	3	4	5	6	7	8	9
001	3.18	0.11	0.34	0.46	0.27	0.25	0.41	0.27	0.51
002	2.67	0.18	0.19	0.26	0.19	0.17	0.24	0.20	0.36
			…120 files…						
125	3.46	0.21	0.31	0.34	0.27	0.28	0.44	0.33	0.60
Mean	3.11	0.16	0.22	0.37	0.21	0.23	0.33	0.27	0.47
Std.	0.327	0.042	0.065	0.082	0.038	0.046	0.088	0.053	0.099

**Table 9:** Correctness of verbose diagnostic outcomes of original, uniformized, non-uniform, and pseudo-non-uniform ECGs (123 CSE records).

Diagnostic Outcome	oECG	uECG	nECG	pECG
**correct**	121	117	115	117
**incorrect statement**	1	2	3	3
**incorrect modifier**	1	4	5	3
**total**	123	123	123	123
